# Supplementary material for: The interactome of intact mitochondria by cross-linking mass spectrometry provides evidence for coexisting respiratory supercomplexes
Source: Mol Cell Proteomics. 2017 Dec 8;17(2):216–32. doi: 10.1074/mcp.RA117.000470 (PMC5795388; doi:10.1074/mcp.RA117.000470)
Supplement: Supplemental Data [file supp_RA117.000470_133922_0_supp_23978_fzffwf.zip › spectra_annotation/mito_spectra_annotation/267-1-1-1-10-1.pdf]

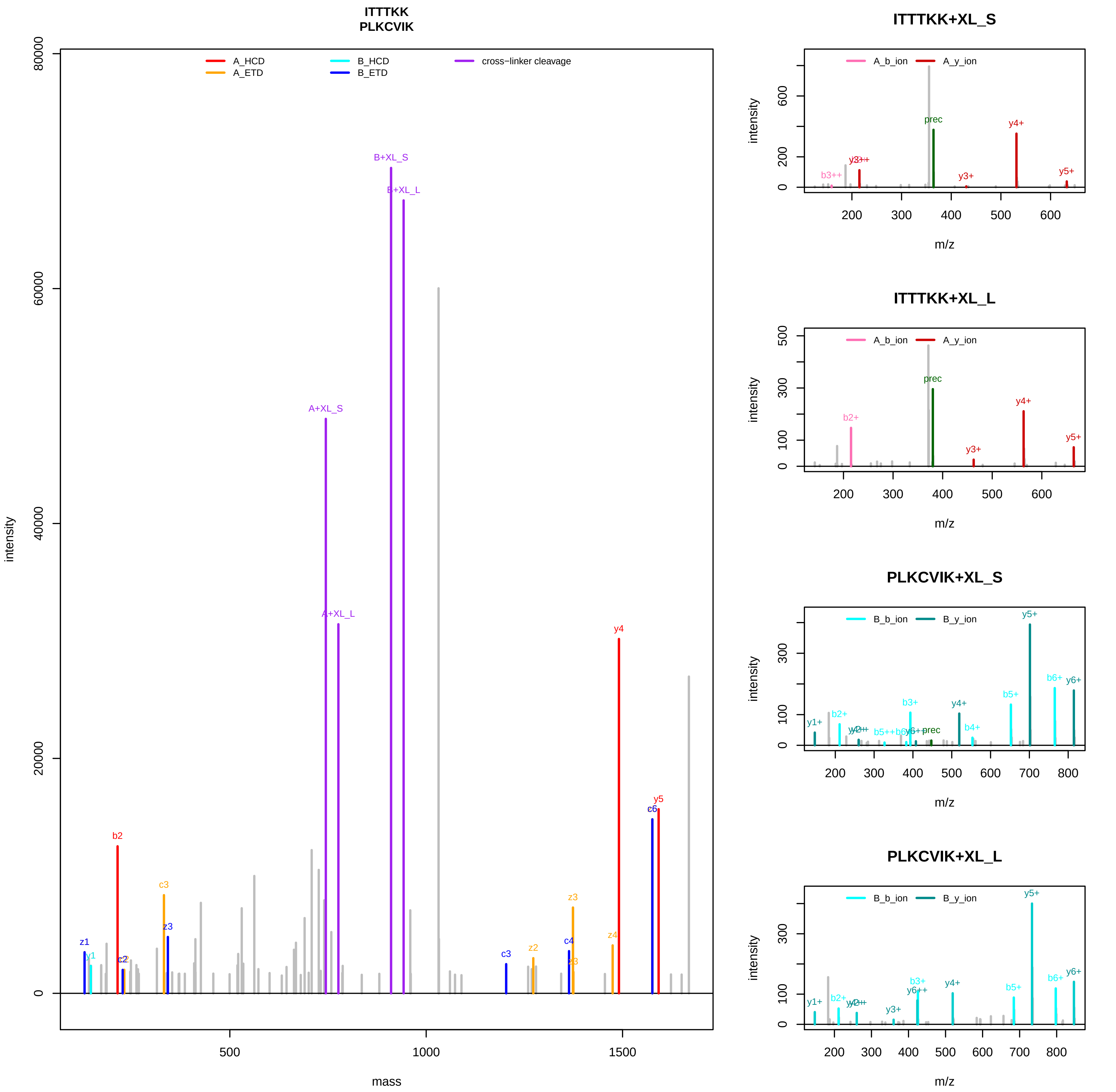

ITTTKK
PLKCVIK
A_HCD
A_ETD
B_HCD
B_ETD
cross−linker cleavage
B+XL_S
B+XL_L
A+XL_S
A+XL_L
y4
y5
c6
b2
c3
z3
z1
y1
c2
c3
z2
c4
z4
intensity
mass
ITTTKK+XL_S
A_b_ion
A_y_ion
prec
y4+
b3++
y3++
y3+
y5+
m/z
ITTTKK+XL_L
b2+
PLKCVIK+XL_S
B_b_ion
B_y_ion
y5+
b6+
y6+
b5+
b3+
y4+
b4+
b2+
y1+
y2++
b5++
y6++
PLKCVIK+XL_L
b3+
y6++
y3+
